# Non-Vesicular Extracellular Particle (NVEP) Proteomes from Diverse Biological Sources Reveal Specific Marker Composition with Varying Enrichment Levels

**DOI:** 10.3390/biom15111487

**Published:** 2025-10-22

**Authors:** Wasifa Naushad, Bryson C. Okeoma, Carlos Gartner, Yulica Santos-Ortega, Calvin P. H. Vary, Lakmini S. Premadasa, Alessio Noghero, Jack T. Stapleton, Ionita C. Ghiran, Mahesh Mohan, Chioma M. Okeoma

**Affiliations:** 1 Department of Pathology, Microbiology and Immunology Basic Sciences Building, New York Medical College, 15 Dana Road, Rms 327, 328, 328A, Valhalla, NY 10595-1524, USA; wnaushad@nymc.edu (W.N.); bokeoma@nymc.edu (B.C.O.); 2 MaineHealth Institute for Research, Center for Molecular Medicine, Scarborough, ME 04074, USA; carlos.gartner@mainehealth.org (C.G.); heq9zv@virginia.edu (Y.S.-O.); calvin.vary@mainehealth.org (C.P.H.V.); 3 Graduate School of Biomedical Sciences and Engineering, University of Maine, Orono, ME 04469, USA; 4 Southwest National Primate Research Center, Texas Biomedical Research Institute, San Antonio, TX 78227-5302, USA; lpremadasa@txbiomed.org (L.S.P.); mmohan@txbiomed.org (M.M.); 5 Lovelace Biomedical Research Institute, Albuquerque, NM 87108-5127, USA; anoghero@lovelacebiomedical.org; 6 Department of Internal Medicine, Carver College of Medicine, University of Iowa, 200 Hawkins Drive, Iowa City, IA 52242-1109, USA; jack-stapleton@uiowa.edu; 7 Medical Service, Iowa City Veterans Affairs Medical Center, University of Iowa, 604 Highway 6, Iowa City, IA 52246-2208, USA; 8 Department of Anesthesia, Critical Care and Pain Medicine, Beth Israel Deaconess Medical Center, Harvard Medical School, Boston, MA 02115, USA; ighiran@bidmc.harvard.edu

**Keywords:** extracellular vesicles (EVs), non-vesicular extracellular particles (NVEPs), proteomics, markers, capillary Western blot

## Abstract

Extracellular particles (EPs), an umbrella term encompassing membrane-enclosed extracellular vesicles (EVs) and non-vesicular extracellular particles ([NVEPs], previously described as extracellular condensates [ECs]) contain a complex cargo of biomolecules, including DNA, RNA, proteins, and lipids, reflecting the physiological state of their cell of origin. Identifying proteins associated with EPs that regulate host responses to physiological and pathophysiological processes is of critical importance. Here, we report the findings of our study to gain insight into the proteins associated with NVEPs. We used samples from human semen, the rat brain, and the rhesus macaque (RM) brain and blood to assess the physical properties and proteome profiles of NVEPs from these specimens. The results show significant differences in the zeta potential, concentration, and size of NVEPs across different species. We identified 938, 51, and 509 total proteins from NVEPs isolated from rat brain tissues, RM blood, and human seminal plasma, respectively. The species-specific protein networks show distinct biological themes, while the species-conserved protein interactome was identified with six proteins (ALB, CST3, FIBA/FGA, GSTP1, PLMN/PLG, PPIA) associated with NVEPs in all samples. The six NVEP-associated proteins are prone to aggregation and formation of wide, insoluble, unbranched filaments with a cross-beta sheet quaternary structure, such as amyloid fibrils. Protein-to-function analysis indicates that the six identified proteins are linked to the release of dopamine, immune-mediated inflammatory disease, replication of RNA viruses, HIV-HCV co-infection, and inflammation. These interesting findings have created an opportunity to evaluate NVEPs for their potential use as biomarkers of health and disease. Additional in-depth studies are needed to clarify when and how these proteins sustain their physiological role or transition to pathogenic roles.

## 1. Introduction

Extracellular particles (EPs), consisting of extracellular vesicles (EVs) and non-vesicular extracellular particles ([NVEPs] and previously described as extracellular condensates [ECs], contain a complex cargo of biomolecules, including DNA, RNA, proteins, and lipids, reflecting the physiological state of their cell of origin [1]. Additionally, NVEPs also include extracellular DNA in the form of nucleosome particles (DNA wound around histones) or strings of nucleosomes (chromatin), released via endosomal and autophagic pathways independent of EVs [2]. NVEP-associated molecules, including extracellular DNA [2] and RNA [3,4,5,6,7], may have been previously characterized as EV-associated, hence the need for accurate and reproducible isolation systems.

**Figure 1:**
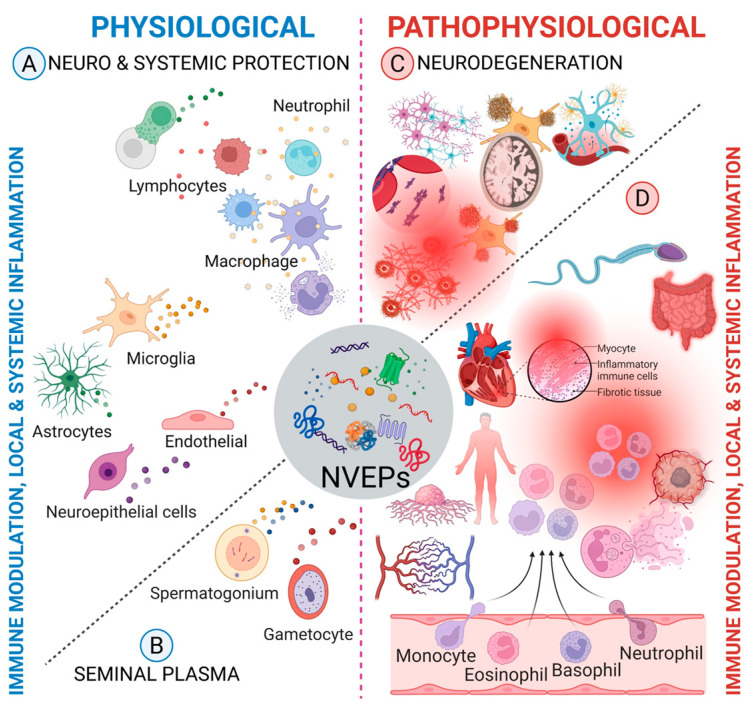
NVEPs are released by different cell types and are present in both tissues and body fluids. They play various tissue-specific roles under physiological (**A**,**B**) and pathophysiological (**C**,**D**) conditions.

Unlike EVs that contain membranes, NVEPs are membraneless condensates [4] known to assemble and incorporate lipids, nucleic acids, and proteins. Like EVs, the type of proteins associated with NVEPs may depend on the status of the parent cell and local environment, which will determine their functions in target cells. While we and others have extensively characterized the protein composition of EVs [8,9], there is a paucity of information on the protein content of NVEPs. However, proteins associated with NVEPs may play key roles in a wide range of normal biological functions and disease processes (Figure 1), including cancers, infectious diseases, cardiovascular diseases, and other chronic diseases such as Alzheimer’s disease (AD), where aggregates/oligomers of amyloid-β peptides form insoluble fibrillar structures that present as amyloid plaques, which have been shown to damage brain cells [10]. As reviewed by Jeppesen et al., 2024 [11], and Li et al., 2025 [12], the stability and biocompatibility of NVEPs make them potential vehicles for intercellular communication and therapeutics delivery.

While NVEPs may interact with host cells through membrane receptors or surface-bound lipoprotein lipases, it remains unclear whether NVEPs and their associated proteins are pathogenic or serve protective functions by facilitating the clearance of misfolded proteins and aggregates. Similarly, the identities and functions of NVEP-specific proteins also remain to be determined. Our group has successfully used particle purification liquid chromatography (PPLC) to efficiently isolate EVs and NVEPs from various biological sources. However, to date, to the best of our knowledge, there have been no NVEP-specific protein markers identified. Our underlying hypothesis is that markers of NVEPs must be common across various species and sample types to have correlative significance. This perspective is premised on the high degree of protein conservation between different model systems and proteomes. In this study, we used unbiased comparative proteomics and bioinformatics approaches to provide a snapshot of extracellular proteins associated with NVEPs isolated from various samples and species, including human semen, the rat brain, and the rhesus macaque (RM) brain and blood

## 2. Materials and Methods

### 2.1. Protein Profiling Across NVEPs Isolated from Different Species and Specimens

#### 2.1.1. Ethical Approval and Specimens

Human semen samples from HIV-negative subjects who consented to participate in this study via written informed consent were collected with the approval of the University of Iowa’s Institutional Review Board (IRB). Brain and blood samples were collected from age- and weight-matched rhesus macaques (RMs) and rats for NVEP protein characterizaton. All experiments were performed in accordance with the approved university guidelines and regulations. The specimens used in this study were collected from different past and ongoing studies supported by NIH grants. The study subjects (human, rat, rhesus macaque) were not infected with any pathogen and not treated with any compound. The plasma portions of blood and semen were processed and stored at −80 °C before being used for isolation of NVEPs. For the brain, the prefrontal cortex (PFC) and basal ganglia (BG) of rats and rhesus macaques (RM), respectivly, were collected. The rat tissues were flash-frozen at −80 °C, while the RM tissues were stored in RNALater prior to use for EC isolation (Table 1).

#### 2.1.2. Isolation of NVEPs

NVEPs were isolated from the specimens listed above using our previously described protocols for blood [4,6,7], the brain [5,13], and semen [4]. EDTA blood plasma samples, seminal plasma samples, or digested brain tissues were subjected to PPLC-based size exclusion chromatography (SEC), as previously described [4,5,6,7,13]. Briefly, samples were liquefied at room temperature for 30 min and centrifuged at 2000× *g* for 10 min and 10,000× *g* for 30 min to remove cellular debris and large vesicles. Samples were then layered on a gravity-packed 7-bead gradient (G-10, G-15, G-25, G-50, G-75, G-100, 2% BCL agarose bead standard) in a 100 cm × 1 cm Econo-Column for blood and seminal plasma or a G-50 50 cm × 0.5 cm Econo-Column for brain tissue digestion. Elution was achieved by gravity using 0.1× Phosphate-Buffered Saline (PBS, Corning, New York, NY, USA). Fractions of 250 µL were collected, and elution profiles were determined by absorbance measurements at 280 nm. The first peak, which contained EVs, was collected and used for different studies. The last peak, which contained NVEPs, was collected, and the NVEPs were stored in aliquots at −80 °C until further analysis.

#### 2.1.3. Characterization

**Figure 2:**
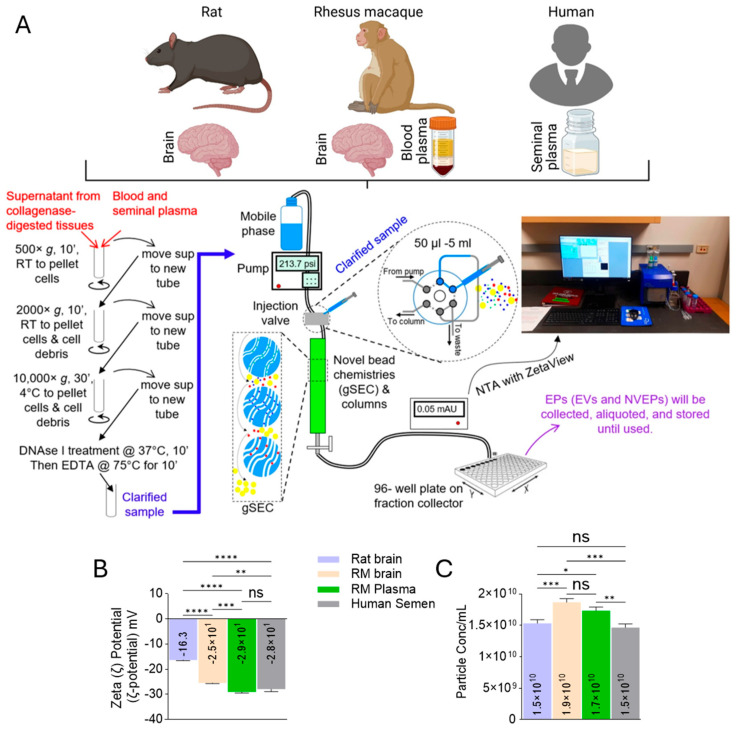
Physicochemical characterization of NVEPs: (**A**) Schematic of the protocol for specimen collection and NVEP isolation. (**B**) Zeta (ζ)-potential of NVEPs. (**C**) Concentration of NVEPs per mL of fluid or mg of tissues. Statistical significance was determined by ordinary one-way ANOVA (Šídák’s multiple-comparison test) and unpaired t test with Welch’s correction. **** *p* < 0.0001, *** *p* 0.0002–*p* 0.0006, ** *p* 0.0029–*p* 0.0051, * *p* 0.0168, ns = non-significant.

A schematic diagram of the workflow for the isolation of BG NVEPs is shown in Figure 2A. Briefly, small chunks of RNALater-stored PFC or BG tissues, ranging from 35 to 118 mg, were finely chopped and digested with collagenase III. Samples were clarified and supernatant was purified on a 50 × 0.5 cm Sephadex G-50 size exclusion column, using a particle purification liquid chromatography (PPLC) system as previously described [4]. Fifty fractions of 200 µL were collected, and the 3D UV-Vis (230–650 nm) fractionation profiles were recorded. A no-tissue collagenase control was used as background. Fractions containing EVs or NVEPs were collected, pooled, and stored in small aliquots at −80 °C. For further characterization, NVEPs were diluted in 0.1× PBS (1/1000). Zeta potential (ζ-potential) and particle concentration measurements were acquired using nanoparticle tracking analysis (ZetaView) as described previously [14].

### 2.2. Nano-Tracking Analysis (NTA)

Specimens were diluted to the appropriate concentration in filtered 0.1X PBS for NTA as previously described by us [15]. Particle concentration and zeta potential (ζ-potential) of the purified specimens were determined (ZetaView PMX110, Particle Metrix, Mebane, NC, USA). The system was calibrated using 100 nm Nanosphere™ size standards (3100A, Thermo Fisher Scientific, Waltham, MA, USA) before acquisition of measurements. The instrument parameters were set up according to recommendations [16]. Samples were then introduced into the chamber (in 1:1000 0.1× DPBS sample dilutions), and measurements were taken in triplicate at multiple positions to ensure accuracy. ZetaView tracked the Brownian motion of particles to determine their size and concentration, utilizing sliding optics to move between focal positions and sample a large volume of the solution. The measured concentration was normalized to the original volume of blood/seminal plasma or tissue weight before dilution and reported in particles/mL/mg. Additionally, the instrument measured the ζ-potential of particles by generating particle drift in an electrical field.

### 2.3. Column Separation

All available size exclusion dextran-based Sephadex^TM^ beads were purchased from GE Healthcare and were as follows: G-10 (17-0010-01), G-15 (170020-01), G-25 fine (17-0032-01), G-50 fine (17-0042-01), G-75 (17-0050-01), and G-100 (17-0060-01). A gradient column was prepared by layering the beads from the smallest to the largest in different Econo-Columns^®^ (Bio-Rad, Hercules, CA, USA) at room temperature by gravity. 1× or 0.1× Phosphate-Buffered Saline (PBS) was used as a mobile phase. Fractions were collected in Greiner UV-Star^®^ 96-well plates using an FC204 fraction collector (Gilson, Middleton, WI, USA), with 6 drops per well. UV-Vis and fluorescence of the fractions were measured using a Synergy H1 plate reader (Biotek, Winooski, VT, USA).

### 2.4. Transmission Electron Microscopy (TEM)

TEM analysis was performed on pooled samples (*n* = 6) as previously described [17]. Briefly, 10 µL of the pooled NVEPs sample was fixed with 1% glutaraldehyde overnight at 4 °C and then absorbed onto a glow-discharged copper grid (Ladd Research) and allowed to sit for 30 s to 15 min. Excess samples were removed with filter paper. The grids were washed three times for 30 s with ultrapure water, stained with 1% uranyl acetate for 3 min, and then allowed to air dry. Images were viewed and collected using a Hitachi HT7700, Tokyo, Japan transmission electron microscope equipped with an XR16M 16-megapixel digital camera system.

### 2.5. Proteomic Analysis

Protein data-independent quantitation (e.g., SWATH) and data-dependent ion spectra libraries were generated as previously described [18,19,20]. Protein identification was determined using the Paragon algorithm [21] in the ProteinPilot v. 5.0.2 (SCIEX) software package, with a <5% false discovery rate (FDR). For quantitative analysis, data-independent mass-spectrometric acquisition using Variable Window SWATH Acquisition [22] was conducted in triplicate and used to determine optimal Q1 isolation windows and increase specificity of detected ions. Peaks were extracted with 95% peptide confidence and 1% FDR. Quantification of SWATH data acquisition was accomplished using MarkerView [23] (SCIEX) and normalized using MLR normalization [24] for SWATH data, as described previously [25]. P-values for fold change were calculated using Welch’s *t*-test in MarkerView (SCIEX).

### 2.6. Statistical Analysis

A proteomics dataset from ProteinPilot (Sciex) was used, along with the inbuilt test within the MarkerView v. 1.3 software, which does not ask for FDR info explicitly. Ingenuity Pathway Analysis (IPA) and STRING web-based bioinformatics inbuilt applications were used for analyses and integration of protein-to-function data. The significance cutoff was set to a fold change (FC) >2.0 or <−2.0 and a *p*-value < 0.05. Ordinary one-way ANOVA, with Šídák’s multiple-comparisons test, or an unpaired *t* test with Welch’s correction were used to assess statistical differences. Details of specific statistics are presented in each figure legend, where the *p*-values are listed. Label-free quantitative analysis was conducted using the relative abundance intensity normalized by the median, using all peptides identified for normalization. The expression analysis was performed considering technical replicates available for each experimental condition or samples following the hypothesis that each group is an independent variable.

### 2.7. Data Mining and Visualization

Interactome networks, pathways, and GO terms were determined using IPA, KEGG and STRING. Heatmaps were plotted with GraphPad Prism (v10.6.1). Venn diagrams were obtained using the Venny platform (v2.1).

### 2.8. Cell Lines

The following reagents were obtained through the NIH HIV Reagent Program, NIAID; NIH: TZM-bl (HRP-8129); ARP-165 HIV-1 infected U937 Cells (U1) contributed by Folks et al., 1989 [26]. The huglia (HC69) microglia cell line was a kind gift from Dr. Jonathan Karn. TZM-bl cells were maintained in DMEM but with 10% FBS (Gibco). U1 cells were grown in RPMI (Gibco-BRL/Life Technologies, Milan, Italy) containing 10% Fetal Bovine Serum (FBS), certified, heat inactivated (Gibco™, Catalog number: A3840001, Waltham, MA, USA), Gibco™ Penicillin-Streptomycin (10,000 U/mL) Catalog number 15140122, Gibco™ Sodium Pyruvate (100 mM) Catalog number 11360070, and Gibco™ L-Glutamine (200 mM) Catalog number 25030081). Huglia (HC69) cells were also maintained in Dulbecco’s modified Eagle medium (DMEM) (Gibco-BRL/Life Technologies) with low glucose, 1.0% fetal bovine serum (FBS), 1.0× glutamine, 50 µg/mL normocin, and 1.0 µM dexamethasone. Prior to the internalization experiment, 10,000 cells were seeded in each well of a 96-well plate and cultured with their respective media in the absence of dexamethasone for huglia cells. Then 24 h later, cells were treated with 5 µg of dye-labeled NVEPs and cultured for another 24 h, with imaging at 6 and 24 h.

### 2.9. Internalization of NVEPs

PBS or NVEPs were stained with 5 µM SYTO™ RNASelect™ or DiR’; DiIC18(7) (1,1′-Dioctadecyl-3,3,3′,3′-Tetramethylindotricarbocyanine Iodide) [4,14,16,27] and then added to cells. Images of the cells were taken at different times, processed (Gen5), and plotted (GraphPad Prism 10.1).

## 3. Results

### 3.1. Isolation and Characterization of the Physical Properties of Non-Vesicular Extracellular Particles (NVEPs) from Blood, Brain, and Semen Samples from Different Species

The workflow for the isolation of NVEPs and their characterization is shown in Figure 2A, at the bottom. We used PPLC [4] to isolate NVEPs from blood, semen, and collagenase-digested brain tissues and separated them from EVs [4]. The NVEPs were collected, aliquoted, and stored at −80 °C until used. Nanoparticle tracking analysis (NTA) was used to evaluate the surface charge of NVEPs, measured as zeta-potential (ζ-potential). In all samples, irrespective of type and source, NVEPs bear negative ζ-potential ranging from −29.1 mV to −16.3 mV (Figure 2B). The ζ-potentials of rat and RM brain tissues were significantly different, with that of the RM brain being more negative (−25.5 mV), while the ζ-potentials of biofluids and blood and seminal plasma were not significantly different. We also acquired data on the NVEPs number while acquiring the ζ-potential data, since there is a minimum concentration (0.1 and 1% *w*/*v*) requirement for successful ζ-potential measurement. We found differences in particle concentrations between the brain tissues and the biofluids (Figure 2C). Although the ζ-potential and concentration of NVEPs were determined, the size was not measured, as the average primary NVEPs size was lower than the 20 nm detection limit of the ZetaView PMX 110 (https://www.particle-metrix.com/pages/brochures-datasheets, accessed on 6 January 2025) with the standard laser configuration, therefore preventing the acquisition of accurate and reproducible data. It is also likely that the limitation of ZetaView for size measurement and the aggregation-prone nature of NVEPs may affect the ζ-potential and concentration data.

### 3.2. NEVP Particles Are Amembranous and Devoid of Tetraspanins

**Figure 3:**
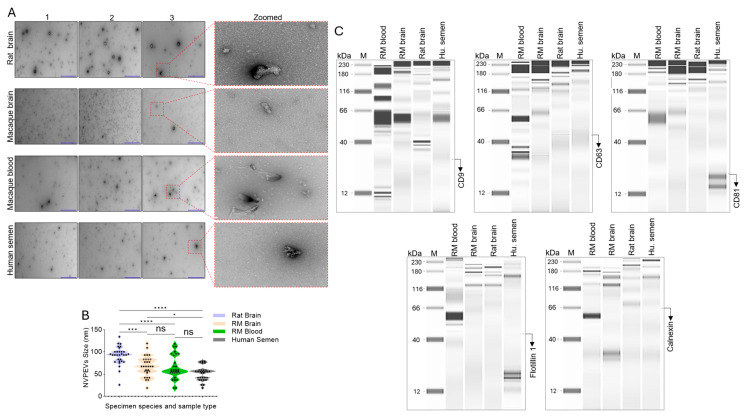
NEVPs are amembranous and devoid of tetraspanins: (**A**) Representative transmission electron microscopic (TEM) of NVEPs. Purple horizontal lines = scale bar = 1.0 µm. Schematic of protocol from the species to the specimens and the isolation method. (**B**) Size of NVEPs measured by TEM (n = 30). Zeta (ζ)-potential of NVEPs. (**C**) Capillary Western blot of markers of NVEPs—CD9, CD63, CD81, Flotillin 1, and calnexin, which was used as a negative control (original images can be found in Figure S1). Error bars represent standard error of the mean. Statistical significance was determined by an unpaired *t* test with Welch’s correction. **** *p* < 0.0001, *** *p* 0.0002–*p* 0.0006, * *p* 0.0168, ns = non-significant. Hu. Semen = human semen.

To assess the morphological differences between the NVEPs, pooled (n = 3) samples were subjected to negative-stain transmission electron microscopy (TEM). The TEM data showed the absence of membrane-containing EVs, an indication that the isolation was successful and that NVEPs were separated from EVs ( Figure 3A and Figure S1A). As revealed by TEM, the NVEPs fraction is enriched in membraneless particles (Figure 3A, red boxes) that range in size from 26 nm to 134 nm, 19 nm to 119 nm, 19 nm to 118 nm, and 19 nm to 79 nm for the rat brain, the RM brain, RM blood, and human semen, respectively (Figure 3B). Details of the descriptive statistics for each type of NVEPs are shown in Table 2, where the mean size of each sample type is 91.63 nm, 67.03 nm, 64.83 nm, and 51.6 nm for the rat brain, the RM brain, RM blood, and human semen, respectively. Similarly, the coefficient of variation was variable. The lower the value of the coefficient of variation, as in the rat brain NVEPs, the more precise the size measurement around the mean, while the greater the coefficient of variation, as in RM blood, the greater the level of dispersion of the size of NVEPs around the mean (Table 2). It is important to note that NVEPs aggregate over time, resulting in sizes differing from the primary size immediately after isolation [4,5].

Since the TEM images clearly show that the NVEPs are distinct from EVs because they are irregular membraneless structures, we used capillary Western blot analysis to assess the presence of common biochemical markers for EVs. We found that tetraspanins (CD9, CD63, CD81) and Flotillin 1 are absent in NVEPs from all species (Figure 3C). Also absent in the NVEPs from all species is the endoplasmic reticulum (ER) membrane marker calnexin, which is used as an EVs-negative marker (Figure 3C and Figure S1B), suggesting that the NVEPs were free from ER membrane contamination.

### 3.3. Species-Independent Internalization of NVEPs by Human Cells

**Figure 4:**
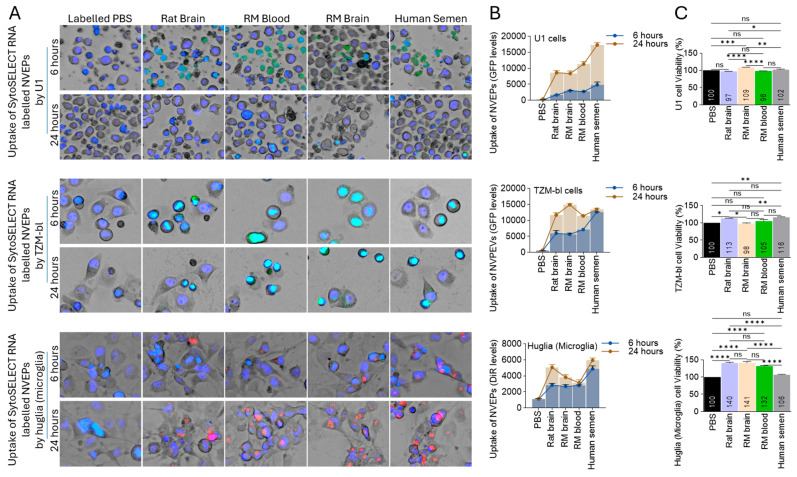
NVEPs from all species were taken up by various human cells and well-tolerated by the cells: (**A**) Representative 10× images of U1 monocytes (top), TZM-bl (middle), and huglia (bottom) incubated with SytoSELECT (green fluorescent)- or DiR (DiIC18(7); 1,1′-dioctadecyl-3,3,3′,3′-tetramethylindotricarbocyanine iodide, red fluorescent)-labeled NVEPs (50 μg) in the presence of NucBlue (a live cell stain, 30 µL/mL), seeded in a glass-bottom 96-well plate, and incubated for 6 and 24 h. (**B**) Quantification of internalized NVEPs at 6 and 24 h. Three fields of view of the images in panel A were quantified with Gen5 software and presented as raw relative florescence units (RFUs). Statistical significance was determined by two-way ANOVA and is presented in Table S1. (**C**) Viability of the different cells treated with NVEPs. Cells treated with PBS were used as a negative control. Statistical significance was determined by ordinary one-way ANOVA (Šídák’s multiple-comparison test). **** *p* < 0.0001, *** *p* 0.0002, ** *p* 0.0043–0.0095, * *p* 0.0154–0.0362, ns = non-significant.

The internalization of labeled NVEPs by three different human cells, including U1 (monocytes), huglia (microglia), and TZM-bl (epithelia), was revealed by green signals for U1 and TZM-bl cells (Figure 4A, top and middle, orange arrows, Figure S2) or red signals for huglia cells (Figure 4A, bottom, orange arrows, Figure S2). The internalization signal was observed in all cell lines at both 6 h and 24 h post-treatment (Figure 4B, Table S1). There were NVEP-dependent differences within the cell lines (Table S1). The reason for the differences in internalization efficiency is not known but may likely be dependent on the interaction between the species-specific molecules on the surface NVEPs and the cell type. The tolerance of the different NVEPs by human cells was assessed using an MTT viability assay. The NVEPs either had no effect on cell viability or they increased the viability of the cells (Figure 4C), an observation that was most prominent in the huglia cells, where NVEPs from the rat brain and the RM brain and blood, but not human semen, significantly increased huglia viability (Figure 4B, bottom). Since the NVEPs were suspended in PBS (0.1×), labeled PBS was used as a control for these studies (Figure 4A–C).

### 3.4. Extracellular Condensates (NVEPs) Are Enriched with Proteins

**Figure 5:**
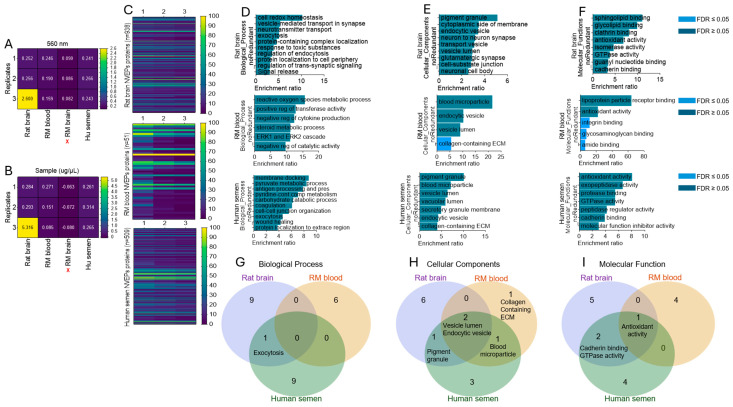
Species-specific extracellular condensate proteomes and their predicted functions: (**A**) Heatmap of 560 nm absorbance spectra of NVEPs. (**B**) Heatmap of NVEP protein concentration (µL). Numbers denote values measured. Red X indicates NVEPs derived from the RM brain not analyzed by MS. (**C**) Heatmap of proteins associated with NVEPs from each species and sample. (**D**–**F**) GO biological processes, cellular components, and molecular functions, respectively. (**G**–**I**) Three-way Venn diagram overlap analysis for biological processes, cellular components, and molecular functions, respectively.

We have shown that NVEPs isolated from RM brains contain microRNA [6,7], but there is a paucity of data on the protein content of NVEPs. To begin the assessment of the protein profiles of NVEPs, we quantitatively measured total protein levels of NVEPs isolated from various species and of different sample types, as described in Figure 2A. The NVEPs have variable total protein contents, as shown by the absorbance spectra at 560 nm (Figure 5A) and the protein concentration/µL (Figure 5B). We then used liquid chromatography coupled to tandem mass spectrometry (LC-MS/MS) analysis to profile the proteome of NVEPs using equivalent protein concentrations (100 ug of each). The RM brain tissue yielded very low (−0.07 ± 0.008 µg/µL) protein levels (Figure 5A,B, marked with **X**) and was not subjected to proteomic analysis. The MS analysis revealed the presence of 938, 51, and 509 total proteins, respectively, from NVEPs isolated from rat brain tissues, RM blood plasma, and human seminal plasma (Figure 5C, Table S2). The proteome of the NVEPs contains proteins from various families and subcellular locations, including the cytoplasm, the nucleus, the cell membrane, mitochondria, and secreted proteins (Table S3). Very low-level peptide ion signals of tetraspanins, including CD9, CD63, and CD81, which are major components of EVs, were detected in the NVEPs from some specimens (Table S2), with the mean values of CD9 being 6.0, 0.0, and 6.4, respectively, for the rat brain, RM blood, and human semen. This observation, along with Figure 3C, suggests that the presence of tetraspanins in NVEPs may be due to minimal or minor contamination from EVs. The bioinformatics toolkit WEB-based Gene SeT AnaLysis (WebGestalt) [28] was used to explore the NVEPs proteins and predict their potential functions. The topmost gene ontology (GO) biological processes identified were cell redox homeostasis, reactive oxygen species processes, and membrane docking, respectively, for the rat brain, RM blood, and human semen NVEPs (Figure 5D). Also, the topmost GO cellular components were pigment granule for the rat brain and human semen NVEPs and blood microparticle for RM blood NVEPs (Figure 5E), while the topmost GO molecular functions were sphingolipid binding, lipoprotein particle receptor binding, and antioxidant activity for the rat brain, RM blood plasma, and human semen NVEPs (Figure 5F). Of the predictions, none of the biological processes overlapped amongst the specimens, while exocytosis was a common GO biological process between the rat brain and human semen NVEPs proteins (Figure 5G). The vesicle lumen and endocytic vesicles were common GO cellular components amongst all three NVEPs proteomes, while pigment granule were common between the rat brain and human semen NVEPs proteins and blood microparticle were a common element between the RM blood plasma and human semen NVEPs proteins (Figure 5H). Moreover, antioxidant activity was a common GO molecular function amongst all three NVEPs proteomes, while cadherin binding and GTPase activity were common elements between the rat brain and human semen NVEPs proteins (Figure 5I).

### 3.5. Species-Specific Network Properties of Proteins Associated with NVEPs

**Figure 6:**
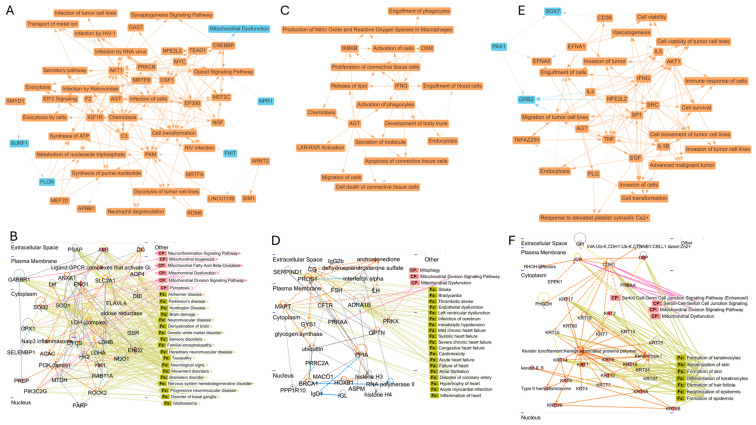
Visualization of species-specific predictive functions of NVEPs-associated proteins. (**A**–**C**) Graphical summary of IPA-predicted functions for proteins associated with NVEPs from the rat brain, RM blood, and human semen, respectively. (**D**–**F**) IPA interactome networks for proteins associated with NVEPs from the rat brain, RM blood, and human semen, respectively, showing proteins and their links to canonical pathway (CP) and diseases and functions (Fx).

To produce comprehensive protein interactome networks of the NVEPs, we applied a strategy that integrated IPA and STRING functional protein association networks to provide insightful knowledge from the gene sets and to reveal the networks and molecular pathways specific to the NVEPs. Our analysis identified the five top major biological themes specific to proteins associated with NVEPs from rat brains (Figure 6A), RM blood (Figure 6B), and human semen (Figure 6C) and their relationships (Table 3).

Moreover, IPA identified 25 networks for proteins associated with rat brain NVEPs. Network 2, containing 26 focused molecules, was linked to neurological diseases, organizational injury and abnormalities, and psychological disorders; the canonical pathway was linked to neuroinflammation, mitochondrial processes, and proptosis; and disease and function were associated with neurological diseases (Figure 6D). Search Tool for Recurring Instances of Neighboring Genes (STRING) (https://string-db.org/, accessed on 6 January 2025) enrichment analysis revealed molecules in the PPI (Figure S3A), with the Kyoto Encyclopedia of Genes and Genomes (KEGG) pathway associated with energy metabolism (Figure S3B). The top five IPA networks for rat brains were merged, and SOD1, which plays roles in mineralization, cell viability, proliferation, apoptosis, number, growth, aggregation, activation, cell death, and formation, was the hub molecule (Figure S3C). Proteins associated with NVEPs from RM blood have links with eight IPA networks, with Network 1 (13 focused molecules) being linked to cancer, cardiovascular disease, organismal injury, and abnormalities; canonical pathways being linked to mitochondrial processes; and disease and function being associated with multiple conditions, including neurological and cardiovascular dysfunction (Figure 6E). Molecules in the PPI network were identified using STRING analysis (Figure S3D), with KEGG pathways associated with glucose metabolism (Figure S3E). Merging the top five IPA networks for RM blood revealed NF-kB (complex), which plays important roles in apoptosis, cell activation, permeability, viability, differentiation, growth, proliferation, survival, and adhesion, as the hub molecule (Figure S3F). Human semen proteins associated with NVEPs produced 25 networks using IPA (Figure 6). Network 1, with 27 focused molecules, was linked to cellular assembly, organization, function, and maintenance, as well as embryonic development; the canonical pathway was linked to spermatogenesis and mitochondrial processes, while disease and function were associated with keratinization (Figure 6F). STRING enrichment analysis revealed molecules in the PPI (Figure S3G), with the KEGG pathway being associated with Staphylococcus aureus infection and estrogen signaling (Figure S3H). The top five IPA networks for human semen were merged, and the immunoglobulin complex, which plays roles in cell proliferation, adhesion/attachment/binding, death/killing/apoptosis, viability, and growth, represented the hub molecule (Figure S3I). These analyses provide insight into and make a case for studying gene sets associated with NVEP- type-specific networks and their potential context-specific role in the host.

### 3.6. Protein–Protein Interactome (PPI) Network and Functional Enrichment of Common Human–Rat–Rhesus Proteins Associated with NVEPs

**Figure 7:**
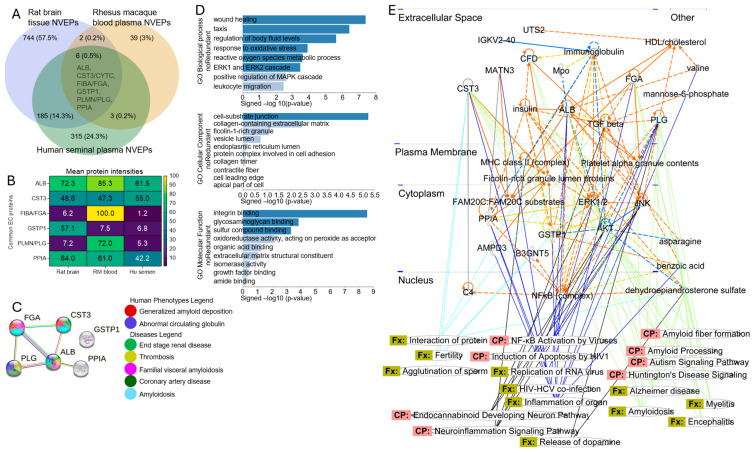
Visualization of conserved functions of six NVEP-associated proteins. (**A**) Three-way Venn diagram overlap analysis for identification of six NVEP-associated proteins showing percentage overlap and the six proteins. (**B**) Heatmap showing levels of six proteins. (**C**) String-based PPI analysis of six proteins. (**D**) GO biological processes, cellular components, and molecular functions, top to bottom, respectively. (**E**) IPA interactome networks for six NVEP-associated proteins showing proteins and their links to canonical pathways (CP), diseases, and functions (Fx).

Here, we identified proteins common to two or all three species and specimen types using Venn diagram overlap analysis (Figure 7A, Table S3). The percentage of proteins common between the rat brain and human semen NVEPs is the largest at ~14% (n = 185). The overlap between the rat brain versus RM blood is 0.2% (n = 2), while that between human semen and RM blood is also 0.2% (n = 3) (Figure 7A, Table S3). Interestingly, six (0.5%) proteins (ALB, CST3, FIBA/FGA, GSTP1, PLMN/PLG, and PPIA) were conserved amongst all species and specimens (Figure 7A, Table S3). The levels of the six common proteins associated with the NVEPs can be visualized on the heatmap (Figure 7B). Using STRING analysis for protein-to-phenotype assessment of the six conserved proteins revealed the disease processes and pathways associated with amyloid buildup, circulating globulin, and chronic diseases, including renal and coronary diseases (Figure 7C). WEB-based Gene SeT AnaLysis Toolkit (WebGestalt) gene ontology (GO) identified the top biological processes associated with cell movement and the regulation of body fluid levels; cellular components suggestive of the cell–substrate junction and the collagen-containing extracellular matrix; and molecular functions associated with integrin and glycosaminoglycan binding (Figure 7D). IPA for functional and canonical pathway networks predicted that the six common proteins potentially participate in neurological (nervous system) disease, NF-kB signaling, virus replication, release of dopamine, and fertility (Figure 7E).

### 3.7. Six NVEP-Associated Proteins as Markers of NVEPs

**Figure 8:**
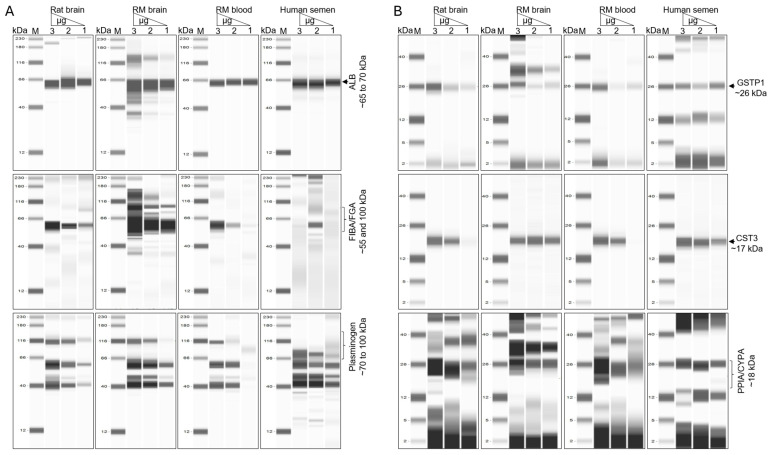
Validation of the levels of six NVEP-associated proteins. (**A**,**B**) Capillary Western blot analysis of high- and low-molecular-weight proteins, respectively (original images can be found in Figure S4).

The levels of the six proteins (ALB, CST3, FIBA/FGA, GSTP1, PLMN/PLG, and PPIA) that were common amongst all NVEPs from the different specimens and species identified to be enriched in NVEPs were analyzed for validation using the Jess^TM^ Simple Western system. The proteins were enriched in NVEPs and were detected in increasing concentrations from 0.8 µg to 1.6 µg and 2.4 µg (Figure 8A,B and Figure S4). The levels of the high-molecular-weight proteins ALB, FIBA/FGA, and plasminogen are presented in Figure 8A, while the levels of the low-molecular-weight proteins GSTP1, PLMN/PLG, and PPIA are presented in Figure 8B. All six proteins identified by MS were validated, suggesting that ALB, CST3, FIBA/FGA, GSTP1, PLMN/PLG, and PPIA may be conserved markers of NVEPs.

## 4. Discussion

In this study, we analyzed the proteome of NVEPs isolated from three different specimens obtained from three distinct species, including brain tissues from rats and RMs, blood plasma from RMs, and seminal plasma from humans. TEM analysis showed that the NVEPs from these specimens were efficiently separated from EVs. The membraneless structures of NVEPs were of different shapes and sizes. Their surface charges measured through ζ-potential, were also different. The differences in ζ-potential were the first indication that the molecular compositions of the NVEPs may be different.

Although equal concentrations (100 µg protein weight) of lysates containing NVEPs from different specimens were used, the RM brain tissues exhibited very low protein content, precluding MS analysis. Similar to NVEPs isolated from RM brain tissues, NVEPs isolated from RM blood also yielded low protein content. The number of proteins identified in NVEPs from RM blood was the lowest at 51 proteins, followed by human seminal plasma at 509 proteins, and then 938 proteins from rat brain tissue. From these data on protein concentrations and the number of proteins identified through MS study, it is evident that the total content and number of proteins associated with isolated NVEPs are variable.

MS analysis identified proteins that are distinct and those that are common amongst the NVEPs. The percentage overlap of proteins associated with NVEPs between the rat brain and human semen NVEPs proteomes is the largest at ~14%, followed by the overlap between the rat brain and RM blood or human semen and RM blood, both of which are 0.2%. Unsurprisingly, the overlap of proteins associated with NVEPs amongst the three species and three sample types from all model systems increased to 0.5%. Venn overlap analysis identified ALB, CST3, FIBA/FGA, GSTP1, PLMN/PLG, and PPIA as being common to all three tissues, and Western blot validated these proteins as conserved amongst NVEPs.

It is interesting to note that the regulation of IGF transport and uptake by IGFBPs in response to elevated platelet cytosolic Ca^2+^, post-translational protein phosphorylation, acute-phase response signaling, and the coagulation system are top canonical pathways linked to the six common proteins associated with NVEPs. The NVEPs are also predicted to be involved in wound healing, chemotaxis, regulation of body fluid levels, response to oxidative stress, reactive oxygen species, ERK1/2 cascade, positive regulation of MAPK cascade, and leukocyte migration. Also predicted to be associated with the proteomes of NVEPs were different diseases, of which the top five include end-stage renal disease, thrombosis, familial visceral amyloidosis, coronary artery disease, and amyloidosis.

The association between the six proteins contained in NVEPs is significant as some of the proteins have physiological or pathophysiological functional roles. For instance, ALB (albumin) is present in body fluids, tissues, and EVs, and in this study, it was shown to be a part of NVEPs. Human serum albumin is the most abundant protein in human blood plasma [29]. Physiologically, ALB regulates blood plasma colloid osmotic pressure and functions as a carrier protein for various endogenous molecules, such as hormones, fatty acids, and metabolites, as well as exogenous drugs, alongside playing a role in neutralizing free radicals. ALB also exhibits esterase-like activity with broad substrate specificity, binds endogenous and exogenous compounds, including inflammatory molecules, and regulates inflammatory and septic immune responses [30,31]. Additionally, lower serum albumin is associated with higher inflammation and carotid atherosclerosis in subjects with HIV under suppressive anti-retroviral therapy [32]. Therefore, the presence of ALB in NVEPs could have physiological ramifications.

Another of the six NVEP-associated proteins is CST3, which is an inhibitor of cysteine proteinases. CST3 regulates the activity of cathepsin S, a protease with central regulatory functions in retinal pigment epithelial cells. CST3 is found in high levels in saliva, tears, seminal plasma, and all cellular types. It is interesting that CST3 is associated with NVEPs because this protein is linked to serious pathophysiology, including cerebral amyloid angiopathy, macular degeneration, and age-related macular degeneration-11 (ARMD11) [33,34]. Remarkably, CST3 expression has been shown to be elevated in the brains of patients with multiple sclerosis [35].

The *FIBA/FGA* gene makes fibrinogen A alpha (Aα), another of the six NVEP-associated proteins. Fibrinogen consists of three homologous chains—α, β, and γ—which are covalently assembled into α2β2γ2. Mutations in the *FIBA/FGA* gene or abnormalities in fibrin(ogen) and fibrinolysis may lead to a variety of disorders with hemorrhagic and thrombotic manifestations, including dysfibrinogenemia [36,37], hypofibrinogenemia [38], afibrinogenemia [39], and renal amyloidosis, characterized by the formation of insoluble fibers due to protein misfolding and deposition in the extracellular matrix [40].

In addition, we found that glutathione S-transferase pi (GSTP1), also known as FAEES3/GST3, is one of the six NVEP-associated protein complexes. GSTP1 is a phase II detoxification enzyme that is enriched in mammalian erythrocytes, present in many tumor tissues, and may function via its non-catalytic, ligand-binding activity. GSTP1 is involved in the formation of glutathione conjugates of both prostaglandin A2 (PGA2) and prostaglandin J2 (PGJ2) [41] and contributes to the formation of novel hepoxilin regioisomers [42]. GSTP1 also negatively regulates CDK5 activity through p25/p35 translocation to prevent neurodegeneration [42].

The PLMN/PLG (plasminogen) protein is a 92–94 kD serine protease synthesized and secreted by the liver [43] that circulates in blood plasma. This inactive protein is converted to an active protease—plasmin—through the activities of plasminogen activators, including tissue plasminogen activator (tPA), urokinase plasminogen activator (uPA), kallikrein, and factor XII (Hageman factor). Plasmin dissolves fibrin-containing blood clots and cleaves fibronectin, thrombospondin, laminin, and von Willebrand factor. Plasmin functions as a mediator of fibrinolysis and a broad-spectrum protease that directly and indirectly degrades various ECM substrates, such as syndecans [44], laminins, fibronectin [45], vascular cell adhesion protein 1 [46], and vitronectin [47]. These activities of plasmin may play roles in physiologic and pathophysiologic processes, which may include fibrinolysis, signaling pathways and intracellular signaling, cell migration, invasion, wound healing, inflammation, myogenesis, muscle regeneration, neurite outgrowth, oncogenesis, and fibrinolysis [48,49].

Moreover, peptidylprolyl isomerase A (PPIA), also known as PPIase A, cyclophilin A (CYPA), or rotamase A, is another of the six NVEP-associated proteins. Members of the PPIA family possess peptidyl-prolyl cis/trans isomerase activity (PPIAses) that catalyzes the cis/trans isomerization of prolyl-peptide bonds involved in protein folding [50]. PPIA binds calcium and plays a critical role in the pathogenesis of various viral (influenza, hepatitis C virus, coronavirus, and HIV) diseases [51,52]. PPIA interacts with HIV Gag (p55, p24) and Vpr, binds HIV Gag, and has been successfully detected in HIV virions [53]; therefore, it may play a role in HIV infection [53,54,55].

With respect to mediating intercellular communication, although scaffold proteins typically found in EVs, such as tetraspanins that act as molecular hubs for the docking and transport of multiple proteins, are absent in NVEPs, FIBA/FGA is one of the six proteins common to the NVEPs. FIBA/FGA is a precursor to fibrin that forms the structural scaffolds for blood clotting and the matrix for cell adhesion and migration, with functional roles in blood clotting and wound healing. The other proteins, including ALB, CST3, and PPIA/CYPA, have functional features that include serving as hubs that bind multiple cellular proteins to promote intercellular signaling. Plasminogen and GSTP1 may function as proteolytic and detoxification factors, respectively.

Whether or not the six NVEP-associated proteins are pathological or non-pathological is yet to be determined. That being said, ALB, CST3, FIBA/FGA, and GSTP1 (FAEES3/GST3) are proteins that are prone to the aggregation and formation of amyloid fibrils. Such proteins may form wide, insoluble, unbranched filaments with a cross-beta sheet quaternary structure, such as amyloid fibrils. Evidence now suggests that both pathological and functional amyloids are present in all forms of life [56,57,58,59,60,61,62], and their formation may be mediated and regulated by cellular processes. While functional amyloids play roles in diverse biological functions [56,57,58,59,60,61,62], including acting as structural scaffolds, regulatory mechanisms, and storage mechanisms, pathological amyloids are involved in abnormal protein deposition, or amyloidosis, as seen in Alzheimer’s or Parkinson’s diseases [63,64,65,66,67]. Based on these findings, there is increased curiosity as to the potential application of NVEPs in the search for biomarkers and the management of health and disease.

It is noteworthy that all the specimens used in this study were frozen at –80 °C and thawed prior to processing. It is unknown whether the freeze–thaw process affected the integrity of NVEPs or the cargo associated with the NVEPs. Nonetheless, we have previously shown that long-term storage of frozen specimens did not significantly alter the physical properties (morphology, concentration, intensity/size) of EVs [68].

## 5. Conclusions

The six NVEP-associated proteins are prone to the aggregation and formation of wide, insoluble, unbranched filaments with a cross-beta sheet quaternary structure, such as amyloid fibrils, indicating the potential for the formation of functional or pathological amyloids. The findings of this study may facilitate the identification of the biological processes and pathways regulating the biogenesis of NVEPs and the evaluation of NVEP-associated proteins as potential biomarkers or drug targets. Additional in-depth studies are needed to clarify when and how these proteins sustain their physiological role or transition to pathogenic roles.

## Figures and Tables

**Table 1:** Description of specimens.

	Rat Brain (PFC)	RM Brain (BG)	RM Blood Plasma	Human Seminal Plasma
Total Number #	6	6	6	6
# used for TEM	6 (pooled into 3)	6 (pooled into 3)	6 (pooled into 3)	6 (pooled into 3)
# used for proteomics	3	3	3	3
# used for capillary Western blot	3	3	3	3
Volume (mL) or weight (mg) of specimens	~100 mg	~100 mg	1 mL	1 mL

**Table 2:** Descriptive statistics.

	*Rat Brain*	*RM Brain*	*RM Blood*	*Human Semen*
*Number of Values*	30	30	30	30
*Minimum (nm)*	26	19	19	19
*25% Percentile (nm)*	79	55.25	53	40.75
*Median (nm)*	93	67	57.5	56
*75% Percentile (nm)*	100	83	93.5	59
*Maximum (nm)*	134	119	118	79
*Range (nm)*	108	119	99	60
*Mean (nm)*	91.63	67.03	64.83	51.6
*Std. Deviation*	20.45	23.98	26.49	15.85
*Std. Error of Mean*	3.733	4.377	4.836	2.894
*Coefficient of Variation*	22.31%	35.77%	40.85%	30.72%

**Table 3:** Biological themes for species-specific NVEPs.

	Rat Brain (Figure 4A)	RM Blood (Figure 4B)	Human Semen (Figure 4C)
Theme 1	Infection and immune response	Immune response and inflammation	Tumor growth and malignancy
Theme 2	Cancer and cell transformation	Apoptosis and cell death	Invasion and metastasis
Theme 3	Metabolism and energy production	Connective tissue regulation and development	Cell viability and cell survival
Theme 4	Chemotaxis and cellular movement	Lipid metabolism and secretion	Immune response
Theme 5	Synaptic signaling and neurobiology	Cell migration and chemotaxis	Cellular motility and migration

## Data Availability

Data is contained within the article and Supplementary Data.

## Supplementary Materials

The following supporting information can be downloaded at: https://www.mdpi.com/article/10.3390/biom15111487/s1, Figure S1: Original full images of (A) the TEM data shown in Figure 3A, (B) the capillary western blot shown in Figure 3C; Figure S2: Original microscopic images of NVEPs internalization shown in Figure 4A; Figure S3: Species-specific PPI and network properties of proteomes of NVEPs of data shown in Figure 4A–C Rat brain NVEPs proteome showing STRING enrichment analysis of PPI molecules, KEGG pathway associated with energy metabolism, and merged 5 top IPA networks respectively. (D–F) Proteins associated with NVEPs from RM blood showing STRING enrichment analysis of PPI molecules, KEGG pathway associated with glucose metabolism, and merged 5 top IPA networks respectively. (G–I) Proteins associated with NVEPs from RM blood showing STRING enrichment analysis of PPI molecules, KEGG pathway associated with Staphylococcus aureus infection and estrogen signaling and merged 5 top IPA networks respectively; Figure S4: Original full images of the capillary western blot shown in Figure 8; Table S1: Statistics for Figure 2—Two-stage linear step-up procedure of Benjamini, Krieger and Yekutieli; Table S2: Total proteins associated with NVEPs from different species and specimens; Table S3: Convergence and divergence of proteins associated with ECs from different species and speciemns.
