# Supplementary material for: Non-Vesicular Extracellular Particle (NVEP) Proteomes from Diverse Biological Sources Reveal Specific Marker Composition with Varying Enrichment Levels
Source: Biomolecules. 2025 Oct 22;15(11):1487. doi: 10.3390/biom15111487 (PMC12650339; doi:10.3390/biom15111487)
Supplement: Supplementary file 1 [file biomolecules-15-01487-s001.zip › Supplemental material_Biomolecules_Final.pdf]

Non-Vesicular Extracellular Particle (NVEP) Proteomes from Diverse Biological Sources Reveal Specific Marker Composition with Varying Enrichment Levels

Wasifa Naushad [1,†], Bryson C. Okeoma [1,†], Carlos Gartner [2], Yulica Santos-Ortega [2,‡], Calvin P.H. Vary [2,3], Lakmini S. Premadasa [4], Alessio Noghero [5], Jack T. Stapleton [6,7], Ionita C. Ghiran [8], Mahesh Mohan [4] and Chioma M. Okeoma [1,5,*]

[1] Department of Pathology, Microbiology and Immunology Basic Sciences Building, New York Medical College, 15 Dana Road, Rms 327, 328, 328A, Valhalla, NY 10595-1524, USA; wnaushad@nymc.edu (W.N.); bokeoma@nymc.edu (B.C.O.); cokeoma@nymc.edu (C.M.O.)
[2] MaineHealth Institute for Research, Center for Molecular Medicine, Scarborough, ME 04074, USA; carlos.gartner@mainehealth.org (C.G.); heq9zv@virginia.edu (Y.S.-O.); calvin.vary@mainehealth.org (C.P.V.)
[3] Graduate School of Biomedical Sciences and Engineering, University of Maine, Orono, ME 04469, USA
[4] Southwest National Primate Research Center, Texas Biomedical Research Institute, San Antonio, TX 78227-5302, USA; lpremadasa@txbiomed.org (L.S.P.); mmohan@txbiomed.org (M.M.)
[5] Lovelace Biomedical Research Institute, Albuquerque, NM 87108-5127, USA; anoghero@lovelacebiomedical.org
[6] Department of Internal Medicine, Carver College of Medicine, University of Iowa, 200 Hawkins Drive, Iowa City, IA 52242-1109, USA; jack-stapleton@uiowa.edu
[7] Medical Service, Iowa City Veterans Affairs Medical Center, University of Iowa, 604 Highway 6, Iowa City, IA 52246-2208, USA
[8] Department of Anesthesia, Critical Care and Pain Medicine, Beth Israel Deaconess Medical Center, Harvard Medical School, Boston, MA 02115, USA; ighiran@bidmc.harvard.edu
* Correspondence: cokeoma@nymc.edu; Tel.:+1 914-594-4722
† These authors contributed equally to this work.
‡ Present address: University of Virginia, 1340 Jefferson Park Ave Charlottesville, VA 22903, U.S.A

Key words: Extracellular Vesicles (EVs), Non-Vesicular Extracellular Particles (NVEPs), proteomics, markers, capillary western blot.

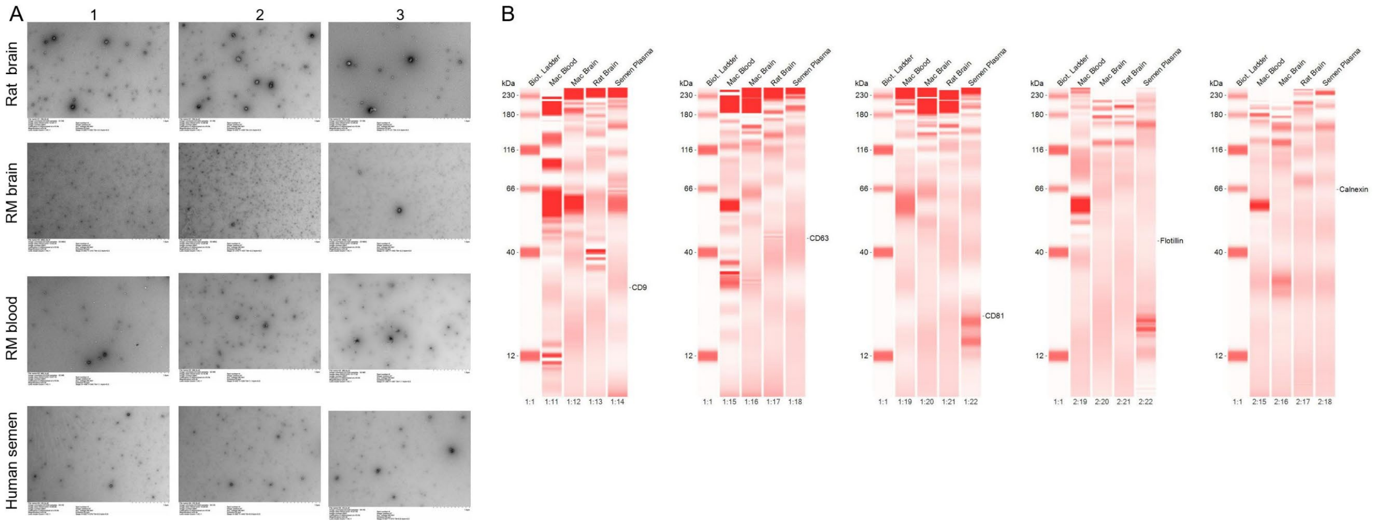

Figure S1: Original full images of (**A**) the TEM data shown in Figure 3A, (**B**) the capillary western blot shown in Figure 3C.

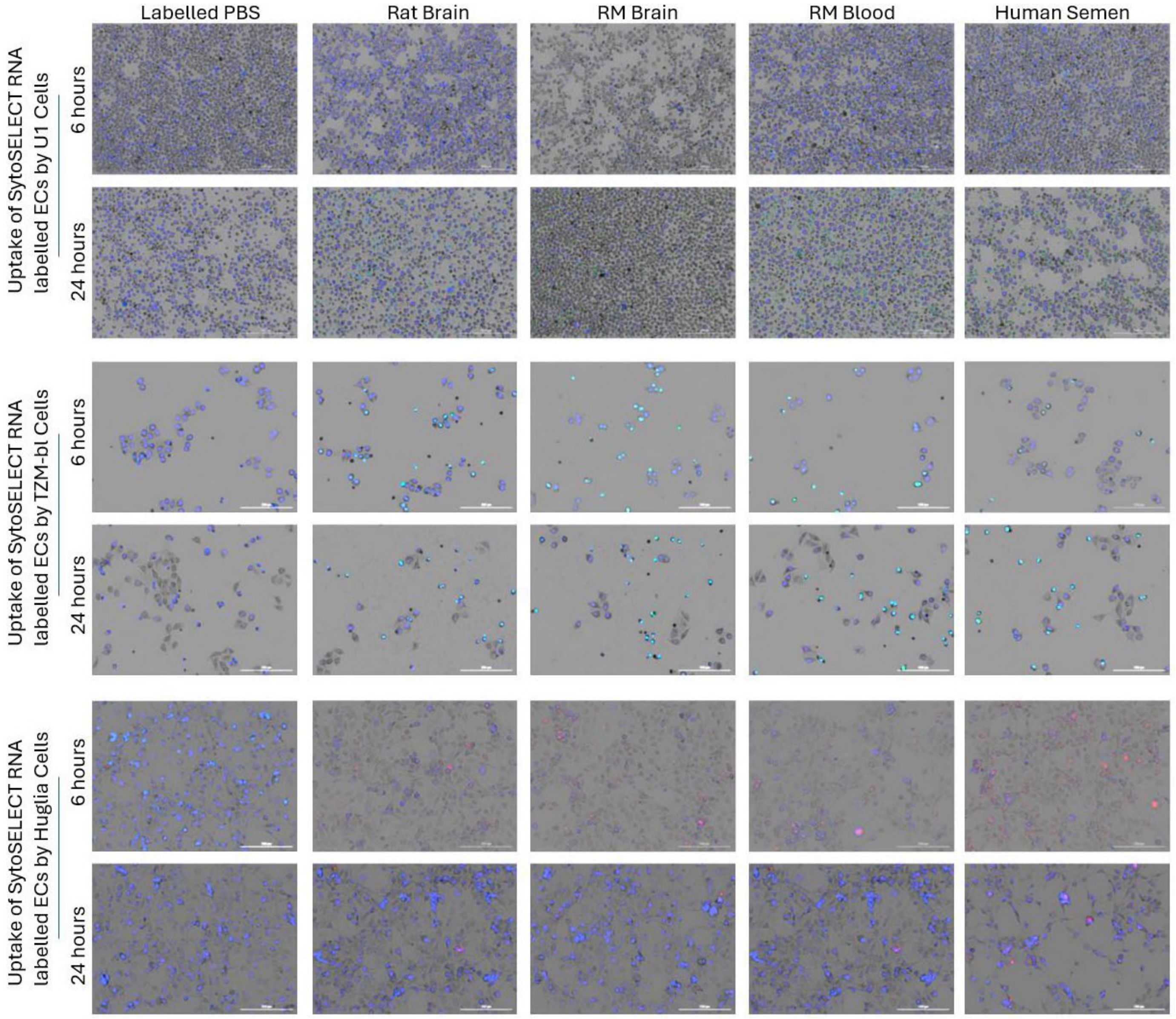

Figure S2: Original microscopic images of NVEPs internalization shown in Figure 4A.

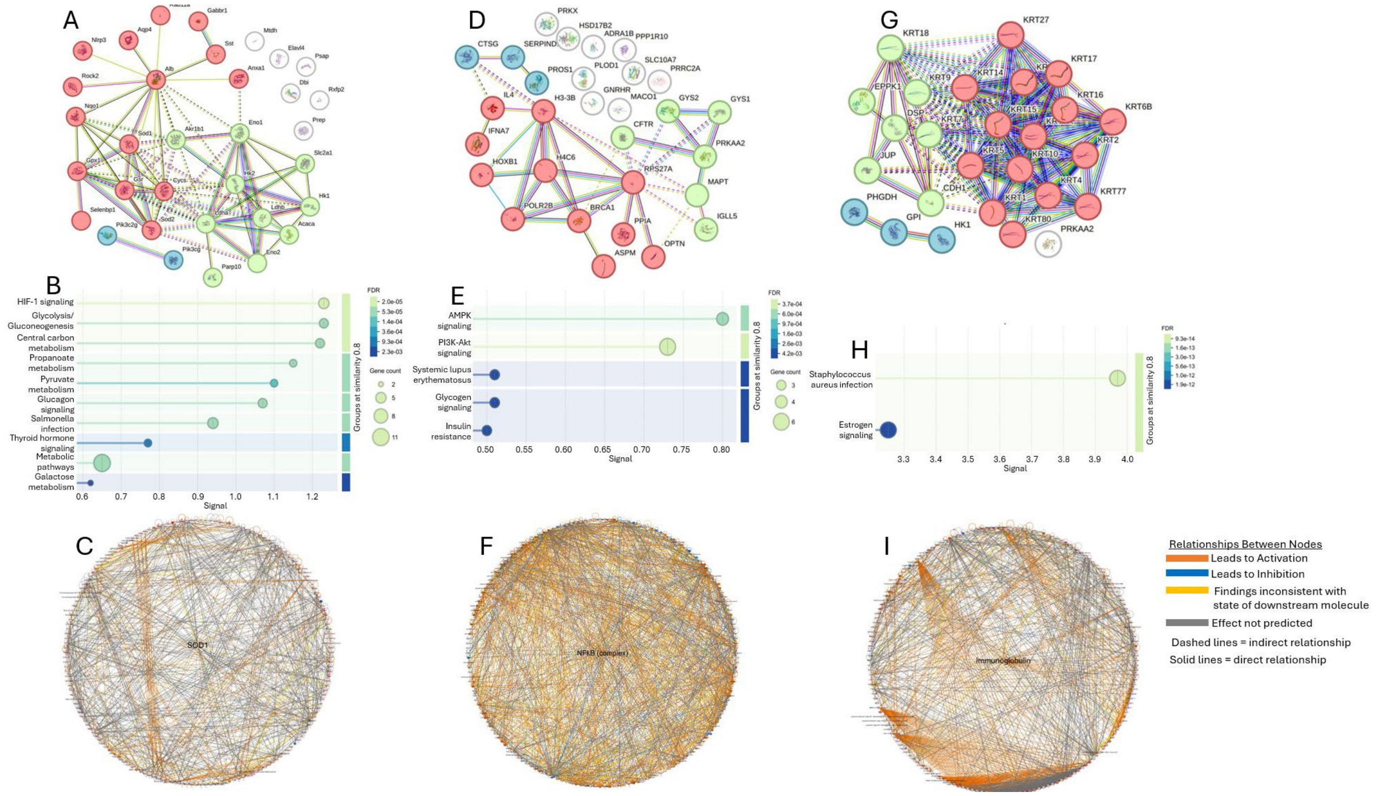

Figure S3: Species-specific PPI and network properties of proteomes of NVEPs of data shown in Fig. 4: (**A to C**) Rat brain NVEPs proteome showing STRING enrichment analysis of PPI molecules, KEGG pathway associated with energy metabolism, and merged 5 top IPA networks respectively. (**D to F**) Proteins associated with NVEPs from RM blood showing STRING enrichment analysis of PPI molecules, KEGG pathway associated with glucose metabolism, and merged 5 top IPA networks respectively. (**G to I**) Proteins associated with NVEPs from RM blood showing STRING enrichment analysis of PPI molecules, KEGG pathway associated with Staphylococcus aureus infection and estrogen signaling and merged 5 top IPA networks respectively.

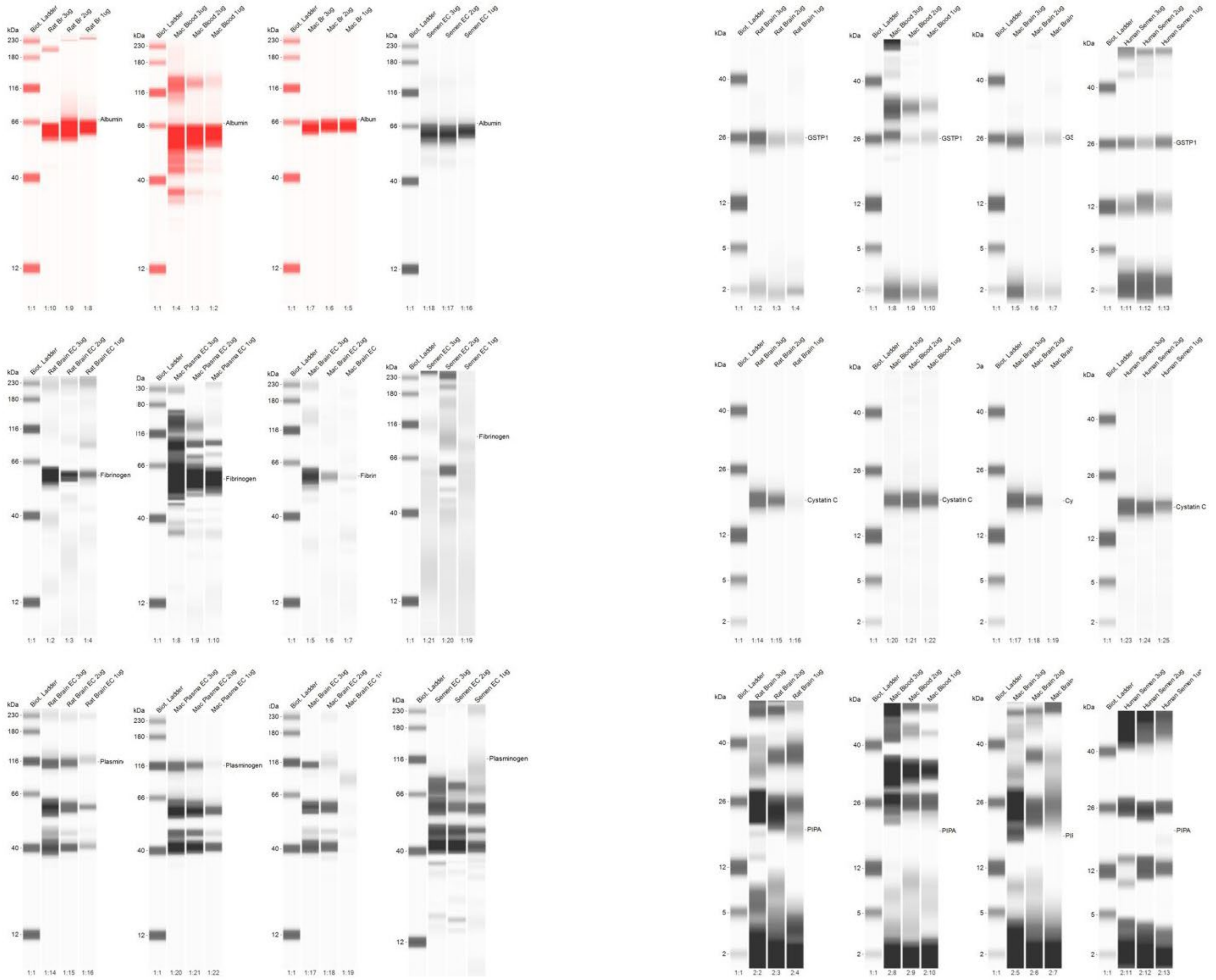

Figure S4: Original full images of the capillary western blot shown in Figure 8.
