# Supplementary material for: Non-Vesicular Extracellular Particle (NVEP) Proteomes from Diverse Biological Sources Reveal Specific Marker Composition with Varying Enrichment Levels
Source: Biomolecules. 2025 Oct 22;15(11):1487. doi: 10.3390/biom15111487 (PMC12650339; doi:10.3390/biom15111487)
Supplement: Supplementary file 1 [file biomolecules-15-01487-s001.zip › Table S1 Statistics for Figure 2 – Two-stage linear step-up procedure of Benjamini, Krieger and Yekutieli.pdf]

Supplemental Table 1: Statistics for Figure 2 – Two-stage linear step-up procedure of Benjamini, Krieger and Yekutieli

Internalization of ECs 6 hours post treatment			Internalization of ECs 24 hours post treatment		
**2-way ANOVA (Tukey's multiple comparisons)**	**Discovery?**	**Individual P Value**	**2-way ANOVA (Tukey's multiple comparisons)**	**Discovery?**	**Individual P Value**
U1 cells (monocytes)					
PBS vs. Rat brain	No	0.0700	*PBS vs. Rat brain*	Yes	<0.0001
PBS vs. RM brain	Yes	0.0017	*PBS vs. RM brain*	Yes	<0.0001
PBS vs. RM blood	Yes	0.0033	*PBS vs. RM blood*	Yes	<0.0001
PBS vs. Human semen	Yes	<0.0001	*PBS vs. Human semen*	Yes	<0.0001
Rat brain vs. RM brain	No	0.1021	*Rat brain vs. RM brain*	No	0.7400
Rat brain vs. RM blood	No	0.1723	*Rat brain vs. RM blood*	Yes	0.0022
Rat brain vs. Human semen	Yes	0.0006	*Rat brain vs. Human semen*	Yes	<0.0001
RM brain vs. RM blood	No	0.7688	*RM brain vs. RM blood*	Yes	0.0010
RM brain vs. Human semen	Yes	0.0292	*RM brain vs. Human semen*	Yes	<0.0001
RM blood vs. Human semen	Yes	0.0154	*RM blood vs. Human semen*	Yes	<0.0001
TZM-bl cells (epithelial cells)					
PBS vs. Rat brain	Yes	<0.0001	*PBS vs. Rat brain*	Yes	<0.0001
PBS vs. RM brain	Yes	<0.0001	*PBS vs. RM brain*	Yes	<0.0001
PBS vs. RM blood	Yes	<0.0001	*PBS vs. RM blood*	Yes	<0.0001
PBS vs. Human semen	Yes	<0.0001	*PBS vs. Human semen*	Yes	<0.0001
Rat brain vs. RM brain	No	0.5498	*Rat brain vs. RM brain*	Yes	<0.0001
Rat brain vs. RM blood	Yes	0.0600	*Rat brain vs. RM blood*	No	0.7208
Rat brain vs. Human semen	Yes	<0.0001	*Rat brain vs. Human semen*	Yes	0.0060
RM brain vs. RM blood	Yes	0.0171	*RM brain vs. RM blood*	Yes	<0.0001
RM brain vs. Human semen	Yes	<0.0001	*RM brain vs. Human semen*	Yes	0.0270
RM blood vs. Human semen	Yes	<0.0001	*RM blood vs. Human semen*	Yes	0.0026
Huglia cells (human microglia. HC69 cells)					
PBS vs. Rat brain	Yes	0.0002	*PBS vs. Rat brain*	Yes	<0.0001
PBS vs. RM brain	Yes	0.0004	*PBS vs. RM brain*	Yes	<0.0001
PBS vs. RM blood	Yes	0.0002	*PBS vs. RM blood*	Yes	<0.0001
PBS vs. Human semen	Yes	<0.0001	*PBS vs. Human semen*	Yes	<0.0001
Rat brain vs. RM brain	No	0.6954	*Rat brain vs. RM brain*	Yes	0.0038
Rat brain vs. RM blood	No	0.9286	*Rat brain vs. RM blood*	Yes	<0.0001
Rat brain vs. Human semen	Yes	<0.0001	*Rat brain vs. Human semen*	Yes	0.0280
RM brain vs. RM blood	No	0.7623	*RM brain vs. RM blood*	Yes	0.0929
RM brain vs. Human semen	Yes	<0.0001	*RM brain vs. Human semen*	Yes	<0.0001
RM blood vs. Human semen	Yes	<0.0001	*RM blood vs. Human semen*	Yes	<0.0001
